# Myocarditis Presenting as ST-Elevation Myocardial Infarction

**DOI:** 10.7759/cureus.47883

**Published:** 2023-10-28

**Authors:** Fahad Hussain, Carmel Moazez, Kathleen Allen, Pamela Hsu, Kathryn Welch, Jennifer Febbo, Mueez Rehman, Mark Garcia

**Affiliations:** 1 Internal Medicine, Northwell Health, Manhasset, USA; 2 Internal Medicine, University of New Mexico School of Medicine, Albuquerque, USA; 3 Cardiology, University of California, Los Angeles, Los Angeles, USA; 4 Cardiology, University of New Mexico School of Medicine, Albuquerque, USA; 5 Radiology, University of New Mexico School of Medicine, Albuquerque, USA

**Keywords:** eosinophilic myocarditis, giant cell myocarditis, cardiac inflammation, cardiac imaging, st-elevation myocardial infarction, myocarditis

## Abstract

When evaluating a patient with ST-segment elevation on ECG and acute chest pain, providers often rapidly arrive at the diagnosis of ST-elevation myocardial infarction (STEMI). As myocardial infarction is deadly and time is of the essence in establishing reperfusion, it is reasonable to place it at the top of the differential. However, doing so should not come at the expense of conducting a thorough clinical evaluation, considering all causes of ST-segment elevation, and creating a comprehensive differential. Myocarditis, in particular, can present similarly to myocardial infarction and misdiagnosis can lead to unnecessary and sometimes harmful interventions such as thrombolytic therapy, vasodilator therapy, or coronary angiography. We present a case of myocarditis mimicking STEMI and discuss diagnosis and treatment of myocarditis.

## Introduction

Myocarditis is defined as inflammation of the cardiac muscle and can have many etiologies including autoimmune, infectious, drug-induced, vaccine-related, or idiopathic. The large variability in presentation, and often subclinical symptoms, make myocarditis a challenging diagnosis. When symptoms are present, they exist on a broad spectrum encompassing fatigue, fever, chest pain, palpitations, dyspnea, lower extremity edema, syncope, and much more. As a result, myocarditis fits many disease profiles and can be misdiagnosed as myocardial infarction, heart failure, or arrhythmia. Definitive diagnosis of myocarditis can only be made through endomyocardial biopsy (EMB), but clinical suspicion can be heightened through history concerning triggers, elevated troponin, ECG abnormalities, structural or functional abnormalities on echocardiogram or cardiac MRI, and absence of atherosclerotic cardiovascular disease on angiogram [1]. Once the diagnosis of myocarditis is made, a detailed history is often necessary to identify the cause. In many cases, the etiology is not able to be determined and management of sequelae, such as heart failure and arrhythmias, is prioritized.

Myocarditis specifically mimicking myocardial infarction is a fairly rare occurrence with an incidence of 0.17 per 1000 man-years [2]. We describe a case of myocarditis initially presenting as ST-elevation myocardial infarction (STEMI).

## Case presentation

**Figure 1:**
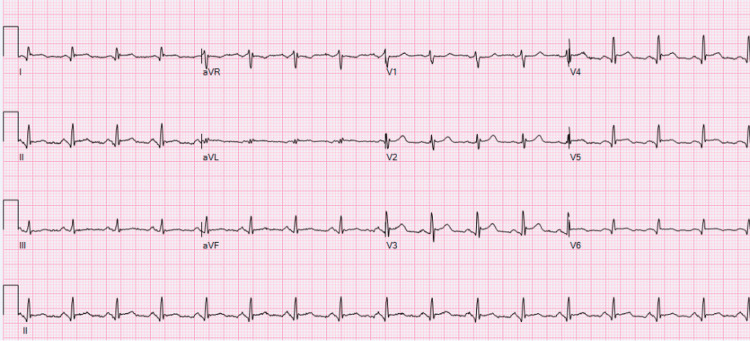
Initial EKG upon presentation to our emergency department

A 50-year-old male with a past medical history of alcohol use disorder presented to an outside hospital with crushing substernal chest pain with radiation to bilateral upper extremities. The patient denied any shortness of breath, palpitations, diaphoresis, lightheadedness, or lower extremity edema. His ECG at the outside hospital reportedly showed ST elevations in leads V2-V6 and he received thrombolytics, aspirin, clopidogrel, heparin, and nitroglycerin before being transferred to the University Hospital for management of a STEMI. When he arrived, he was chest pain-free and his ECG showed ST elevations in leads V2-V6 which did not quite meet STEMI criteria (Figure 1).

**Figure 2:**
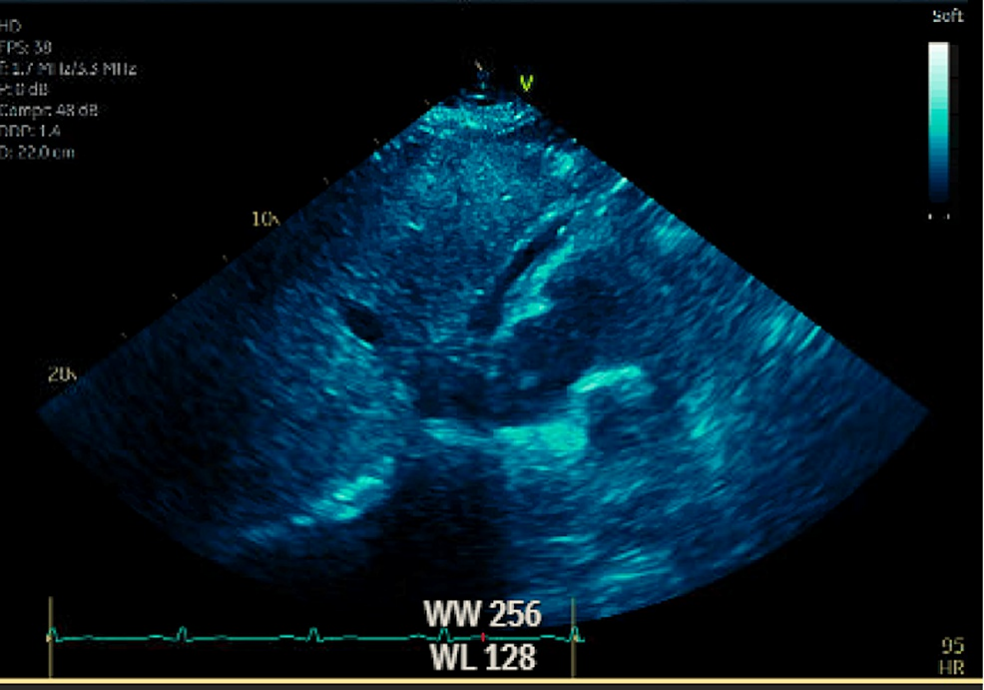
TTE subcostal view showing small anterior pericardial effusion TTE: Transthoracic echocardiography

Labs were significant for troponin greater than 40 ng/l (normal 0-14), B-type natriuretic peptide (BNP) of 3418 pg/ml (normal 0-100 pg/ml), and white blood count of 14700/ul (normal 4,000/ul-11,000/ul). Electrolytes were within normal limits and chest X-ray was unremarkable. Transthoracic echocardiogram showed an ejection fraction of 39%, inferolateral wall hypokinesis, anterolateral wall hypokinesis, inferoapical wall hypokinesis, anteroapical wall hypokinesis, grade 1 diastolic dysfunction, mild pulmonary hypertension, and small pericardial effusion (Figure 2). Thrombus could not be ruled out. Angiogram showed mild luminal irregularities of all vessels, but no obvious culprit lesion. There was some thought that he had an LAD thrombus, lysed by thrombolytics. Cardiac MRI was ordered to work-up alternative pathologies and determine if the patient had a left ventricular (LV) thrombus. 

**Figure 3:**
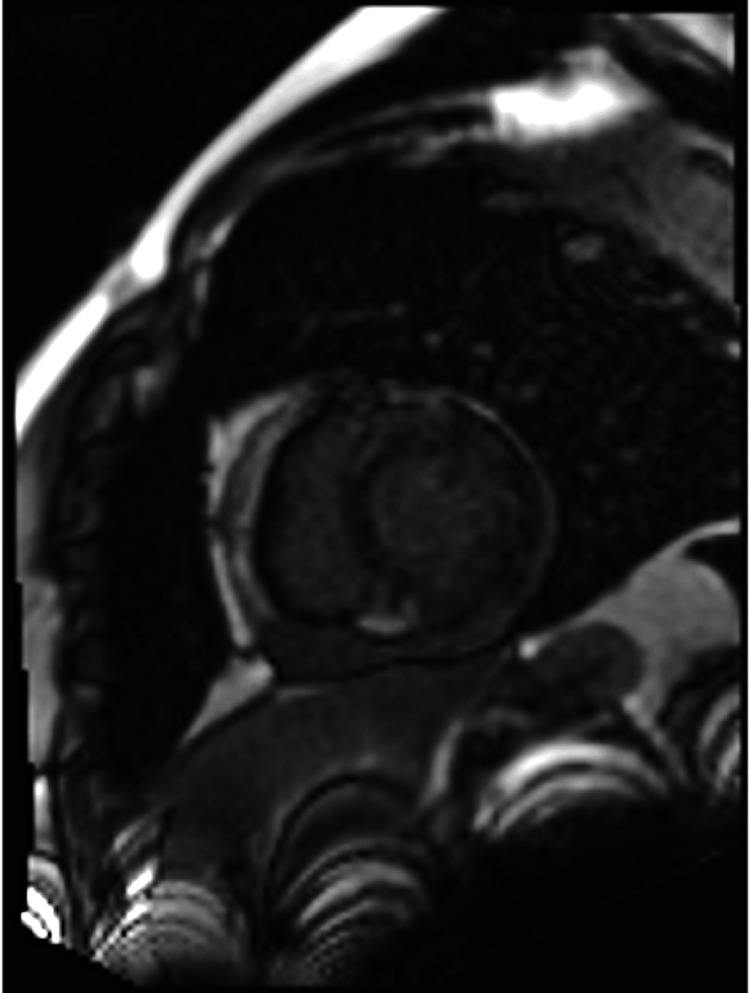
Initial MRI showing late gadolinium enhancement

**Figure 4:**
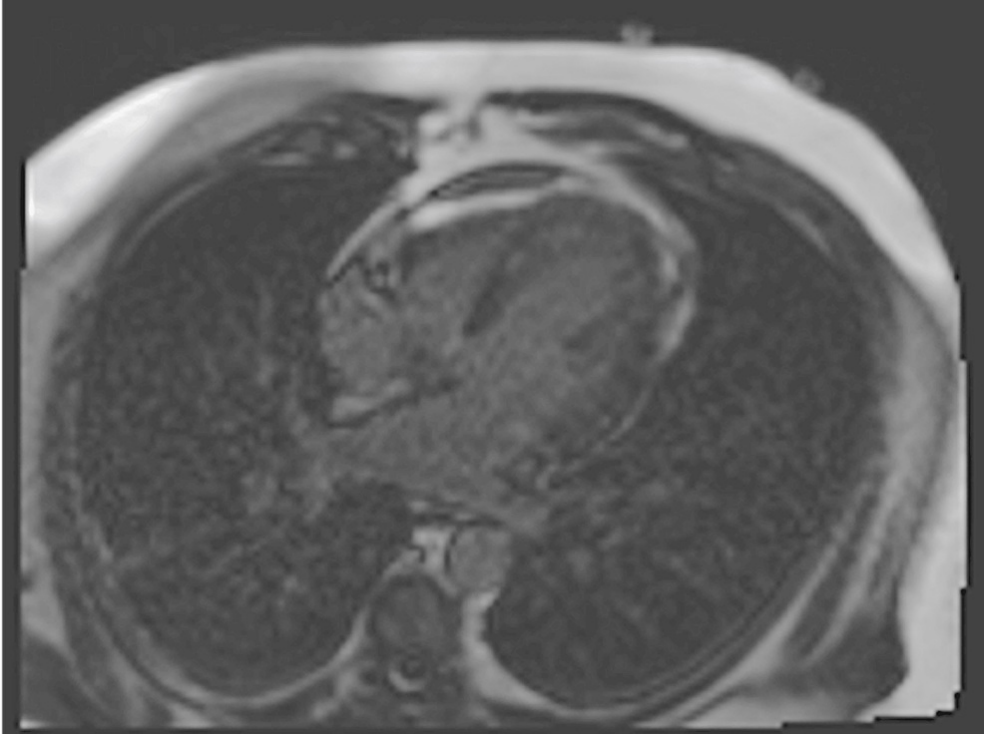
Initial MRI showing late gadolinium enhancement

On the evening of hospital day three, the cardiac MRI was done which showed the greatest degree of delayed gadolinium enhancement in the mid myocardium of the basal and anterior walls of the left ventricle, favoring myocarditis (Figures 3, 4). Additionally, there was moderately decreased left ventricular function, mildly decreased right ventricular function, and an absence of apical left ventricular thrombi. T1 and T2 image mappings were not performed as the study was protocolized for LV thrombus. On hospital day four, the patient developed hypotension and was found to be diaphoretic and clammy. Repeat echocardiogram showed small-to-moderate, pericardial effusion that increased in size anteriorly, without evidence of tamponade. CT angiogram was without evidence of pulmonary embolism but did show bilateral pleural effusions and pulmonary edema. The patient was transferred to the cardiothoracic-vascular intensive care unit for acute decompensated heart failure and concern for cardiogenic shock. He was started on dobutamine for inotropic support and norepinephrine for a possible component of distributive shock. Due to concern for hospital-acquired pneumonia, he was started on vancomycin and cefepime. At this time, there was a higher degree of concern for fulminant myocarditis as the cause of his symptoms.

**Figure 5:**
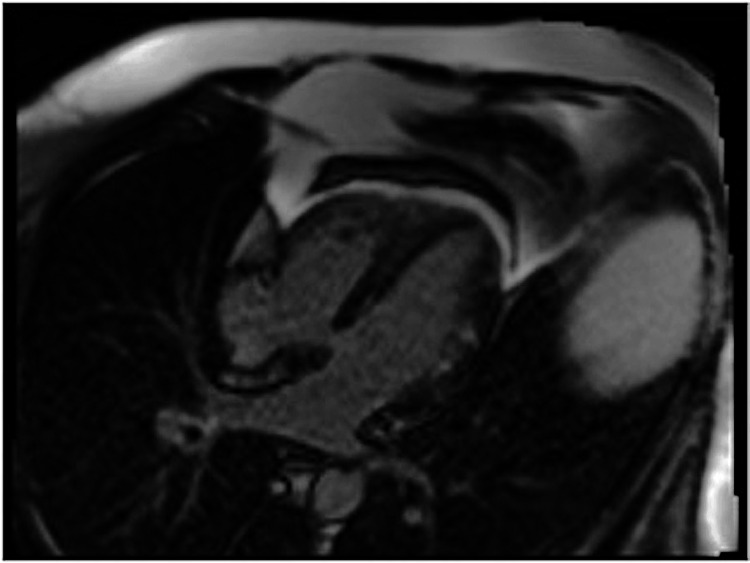
Follow-up MRI 4-chamber view showing multifocal, patchy, mid-myocardial late gadolinium enhancement

**Figure 6:**
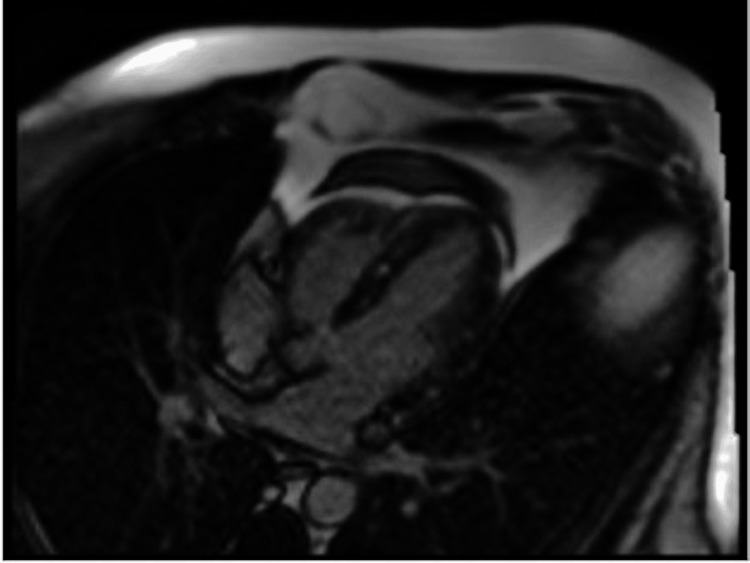
Follow-up MRI 4-chamber view showing multifocal, patchy, mid-myocardial late gadolinium enhancement

**Figure 7:**
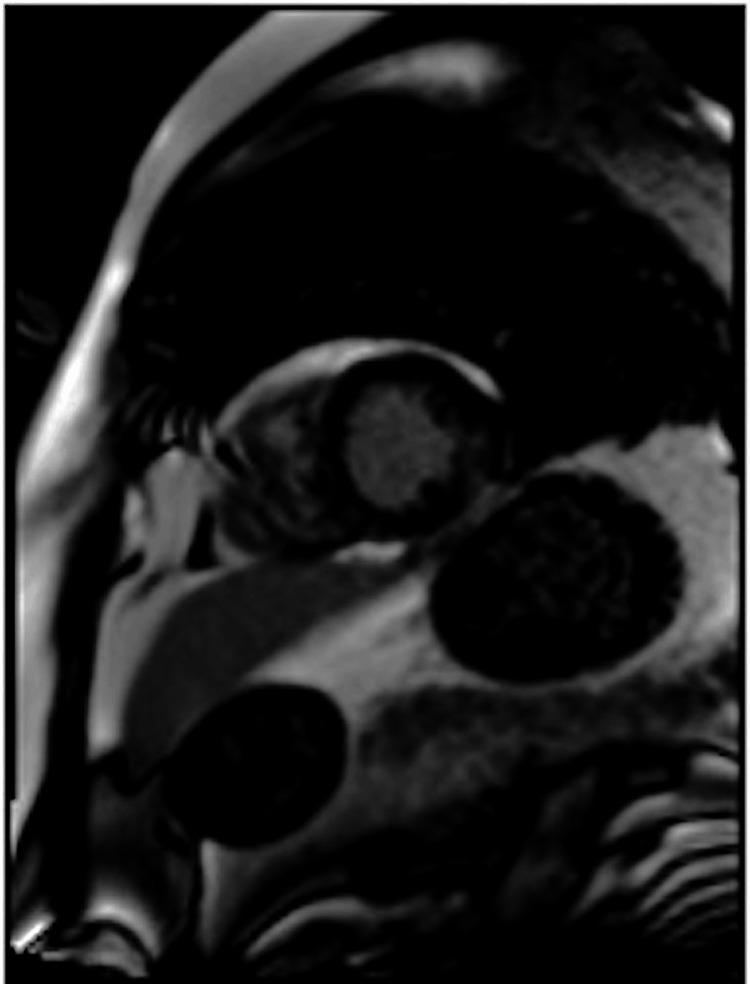
Follow-up MRI short axis view showing multifocal, patchy, mid-myocardial late gadolinium enhancement

The patient underwent EMB and rheumatologic work-up. Infectious disease was consulted to rule out an infectious etiology of myocarditis. Ultimately, the rheumatology and infectious disease work-ups were unremarkable. The EMB was evaluated at Mayo Clinic and was inconclusive. The sample was found to be negative for SARS-CoV-2 RNA. The patient was treated for presumed giant cell vs eosinophilic myocarditis with prednisone and tacrolimus. After nine days in the ICU, he was hemodynamically stable enough to be transferred back to the floor. He had a repeat echocardiogram at the time which showed an ejection fraction of 54.7% and no pericardial effusion. He had a repeat cardiac MRI two weeks after presentation which showed no change in multifocal mid-myocardial late gadolinium enhancement (greatest in the anterior and anterolateral wall), possibly representing a sequela of myocarditis (Figures 5-7). 

Ultimately, the patient was put on goal-directed medical therapy for his heart failure, provided a wearable cardioverter-defibrillator, and discharged home on tacrolimus and a slow prolonged prednisone taper. A repeat cardiac MRI was done after three months, demonstrating a small but improved amount of gadolinium enhancement in the mid-septum and inferolateral walls and an LV ejection fraction of 54%. He was scheduled for close follow-up in the cardiology clinic after discharge and has since been doing well.

## Discussion

Although cases of myocarditis mimicking STEMI are rare, they are certainly represented in the literature [3-5]. In a patient presenting with acute chest pain and ST elevations on ECG, work-up of acute coronary syndrome is the first step. Myocardial infarction can be life-threatening and time is of the essence in reperfusing the myocardium. However, it is important to understand that there are other causes of ST-segment elevation and to conduct a thorough clinical evaluation to create a comprehensive differential. Doing so might save a patient from undergoing unnecessary and potentially harmful interventions such as thrombolytic therapy, vasodilator therapy, or coronary angiography.

Myocarditis may be misdiagnosed as STEMI, on presentation, for a variety of reasons. First, the presentation for myocarditis is highly variable and can overlap significantly with that of a myocardial infarction. Symptoms often include fatigue, fever, chest pain, palpitations, dyspnea, lower extremity edema, and syncope. Oftentimes, as in this case, there are no symptoms except for acute chest pain. Additionally, specific cues in a patient history that might guide a physician to the diagnosis could be sensitive for the patient to discuss (history of drug use, HIV, etc) or may not occur to the patient as something worth mentioning (recent flu-like symptoms, vaccinations, etc). As such, it is the physician's responsibility to obtain a comprehensive history and elicit information specific to the diagnosis. A recent analysis of a case series found that patients with myocarditis mimicking STEMI were characterized by a young age, existence of an infectious prodrome, and elevated inflammatory biomarkers [6]. Lastly, definitive diagnosis of myocarditis is made through EMB, a significantly invasive procedure. Regardless of clinical suspicion, physicians may opt to rule out myocardial infarction with coronary angiography before proceeding to evaluate for myocarditis due to differences in acuity of the conditions as well as the invasiveness of the diagnostics.

Although definitive diagnosis of myocarditis necessitates EMB, it is crucial for physicians to understand the diagnostic criteria for clinically suspected myocarditis. These criteria can serve to increase clinical suspicion of myocarditis based on the presence or absence of certain clinical presentations coupled with non-invasive diagnostic data [1]. By better understanding which patients are at the highest risk for myocarditis, providers can make informed decisions regarding further testing before an invasive intervention such as an EMB. Additionally, cardiac MRI provides non-invasive tissue characterization of the myocardium and can be used to support the diagnosis of myocarditis [1]. Although it can be difficult to differentiate myocarditis from STEMI in some situations, cardiac MRI, comprehensive history-taking, and the diagnostic criteria for clinically suspected myocarditis [1] can all be used to increase suspicion of myocarditis.

After the diagnosis of myocarditis is made, treatment typically consists of general measures to treat sequelae as well as more specific measures tailored to etiology of the myocarditis. Common sequelae of myocarditis include heart failure and arrhythmias. Patients with myocarditis who show signs of heart failure should receive goal-directed medical therapy for acute or chronic heart failure, depending on presentation [1]. Patients can also develop tachyarrhytmias or bradyarrhythmias as a result of myocarditis. These arrhythmias often are not life-threatening and resolve after the acute phase of myocarditis, and, as a result, supportive treatment is generally recommended. Anti-arrhythmic medications are generally reserved for sustained supraventricular tachycardia and non-sustained ventricular arrhythmias, while sustained ventricular arrhythmias are treated with urgent cardioversion [1]. Complete heart block or symptomatic bradycardia can be indications for temporary pacing [1]. Patients with myocarditis who develop symptoms of systemic embolism or acute left-ventricular thrombus can be anticoagulated according to standard criteria for anti-coagulation of patients with atrial fibrillation [1]. Interventions specific to the etiology of myocarditis include immunosuppressive therapy for giant cell myocarditis, sarcoidosis, non-infectious eosinophilic myocarditis, and autoreactive myocarditis [1]. Immunosuppressive regiments can also be used in the treatment of virus-negative, lymphocytic myocarditis although efficacy has not been established by current data [1]. Similarly, although efficacy is unknown, anti-viral agents have been used to treat virus-positive, lymphocytic myocarditis [1]. Although IVIG is another option for treatment of acute myocarditis of autoimmune etiology, there is insufficient data to recommend it at this time [7]. Both general management of myocarditis sequelae and specific therapy targeted to etiology are best practices for comprehensive care of this patient population.

## Conclusions

The patient described in this case presented as a STEMI to a small rural hospital and received thrombolytic therapy. Although it is always important to address life-threatening etiologies of chest pain first, providers can often take non-invasive steps to increase clinical suspicion of other diagnoses before acting. In the case of myocarditis, these steps include utilizing the diagnostic criteria for clinically suspected myocarditis, cardiac MRI, and comprehensive history-taking. Once the diagnosis is confirmed by EMB, management consists of etiology-specific treatment as well as treatment for sequelae of myocarditis as described above.
